# Assessing Symptomatic Hypocalcemia Risk After Total Thyroidectomy: A Prospective Study

**DOI:** 10.1055/s-0043-1777450

**Published:** 2024-02-05

**Authors:** Andro Košec, Ana Gašić, Filip Hergešić, Ivan Rašić, Vesna Košec, Vladimir Bedeković

**Affiliations:** 1 Department of Otorhinolaryngology and Head and Neck Surgery, University Hospital Centre “Sestre milosrdnice,” Zagreb, Croatia; 2 School of Medicine, University of Zagreb, Zagreb, Croatia; 3 Department of Gynecology and Obstetrics, University Hospital Centre “Sestre milosrdnice,” Zagreb, Croatia

**Keywords:** calcium, hypocalcemia, parathyroid hormone, total thyroidectomy, risk assessment

## Abstract

**Introduction** The most common postoperative complication of total thyroidectomy is hypocalcemia, usually monitored using serum parathyroid hormone and calcium values.

**Objective** To identify the most accurate predictors of hypocalcemia, construct a risk assesment algorithm and analyze the impact of using several calcium correction formulas in practice.

**Methods** A prospective, single-center, non-randomized longitudinal cohort study on 205 patients undergoing total thyroidectomy. Parathyroid hormone, serum, and ionized calcium were sampled post-surgery, with the presence of symptomatic or laboratory-verified asymptomatic hypocalcemia designated as primary outcome measures.

**Results** Parathyroid hormone sampled on the first postoperative day was the most sensitive predictor of symptomatic hypocalcemia development (sensitivity 80.22%, cut-off value ≤2.03 pmol/L). A combination of serum calcium and parathyroid concentration sampled on the first postoperative day predicted the development of hypocalcemia during recovery with the highest sensitivity and specificity (94% sensitivity, cut-off ≤2.1 mmol/L, and 89% specificity, cut-off ≤1.55 pmol/L, respectively). The use of algorithms and correction formulas did not improve the accuracy of predicting symptomatic or asymptomatic hypocalcemia.

**Conclusions** The most sensitive predictor of symptomatic hypocalcemia present on the fifth postoperative day was PTH sampled on the first postoperative day. The need for algorithms and correction formulas is limited.

## Introduction

Total thyroidectomy (TT) is a surgical procedure that represents one of the safest procedures in modern surgery, with a short recovery period and low postoperative complication rates. Being the primary surgical treatment option in thyroid malignancy and most benign conditions, increased incidence of newly discovered disease leads to an increase in procedures and several adverse events. The most common postoperative complications are hypocalcemia and recurrent laryngeal nerve injury, transient in most cases. 1 Reports of postoperative hypocalcemia vary significantly due to the heterogeneity of observational studies and the various definitions of hypocalcemia by different authors, with data listing values from 2% to 83%. 2 Total thyroidectomy is always accompanied by short-term dysfunction of the parathyroid glands, which does not necessarily result in postoperative hypocalcemia. 3 Hypocalcemia is most commonly registered when parathyroid hormone (PTH) levels decline >80% concerning preoperative values. 4 The reason for rapid-onset postoperative hypoparathyroidism is the short lifespan (t1/2) of parathyroid hormone and the sensitivity of the parathyroid glands to manipulation. Clinical hypoparathyroidism is defined as biochemical hypoparathyroidism with symptoms and/or signs of hypocalcemia present. 5 Although most hypocalcemia is transient, its occurrence is significant because it leads to prolonged hospitalization, can cause uncomfortable symptoms in patients, reducing their quality of life, and may lead to life-threatening conditions in rare instances. 6 Serum and ionized calcium concentrations, and more recently PTH concentrations, are standardly used to monitor postoperative hypocalcemia. Several studies indicate that early postoperative PTH is the most accurate predictor of the development of symptomatic hypocalcemia after total thyroidectomy, but differing cut-off values and hypocalcemia criteria complicate the direct comparison of data. In addition, some studies recommend using correction formulas such as the Payne correction formula for accurate serum calcium concentration. 6 7 8 9 10 11

This prospective, observational, non-randomized longitudinal cohort study focused on three fundamental problems faced when dealing with assessing post-thyroidectomy hypocalcemia risk. First, identifying the most reliable parameter for assessing both symptomatic and asymptomatic postoperative hypocalcemia and their respective predictive value. Second, developing a simple and practical algorithm for identifying high-risk patients susceptible to postoperative hypocalcemia, requiring daily calcium monitoring and prolonged calcium supplement treatment. Finally, it will analyze the reliability and practicality of using various calcium correction formulas in routine practice.

## Methods

This study included patients who underwent total thyroidectomy in a tertiary referral center from June 2020 to March 2021. A total of 205 patients were included. The study was approved by the Hospital Ethics Committee (EP-12939 / 18–17), following the International Conference on Harmonization principles and the Declaration of Helsinki. The study was pre-registered in the ClinicalTrials.gov online database.

Inclusion criteria were total thyroidectomy regardless of the surgical indication, availability of data on serum albumin, serum calcium, ionized calcium, and PTH, and the presence of hypocalcemia symptoms, as well as signed informed consent forms. Patients with incomplete data, preoperative pathological calcium or PTH levels, and pathological conditions affecting calcium metabolism and parathyroid function were excluded from the study. Data collected included age and sex, diagnosis leading to surgical treatment, whether neck dissection was performed alongside total thyroidectomy, preoperative and postoperative laboratory values (serum calcium, ionized calcium, albumin-corrected calcium, and PTH) during the first five postoperative days, postoperative presence of hypocalcemia symptoms, and need for postoperative calcium replacement therapy.

The primary outcome measures were the presence of hypocalcemia symptoms during the first five postoperative days and the presence of laboratory-verified asymptomatic hypocalcemia on the fifth postoperative day. A secondary outcome measure was the association between the constructed algorithm for assessing hypocalcemia risk on the first postoperative day and hypocalcemia on the fifth postoperative day. Preoperative blood samples for measuring calcium, PTH, and albumin were taken after hospital admission. Postoperative serum PTH, total serum calcium, and ionized calcium were measured one hour after surgery and at 7 am on the first and fifth postoperative days. If hypocalcemia was observed in the patient on the first postoperative day, serum calcium measurements were performed daily. PTH was measured by electrochemiluminescence immunoassay, and serum calcium by the o-cresophthalein complex method, with reference values for PTH ranging from 1.6 to 6.9 pmol/L (15.09- 65.07 pg/mL) and for serum calcium 2.14–2.42 mmol/L (8.0–12.0 mg/dL), respectively. The reference values for ionized calcium were 1.05–1.3 mmol/L (4,2–5,2 mg/dL) and for albumin 34–54 g/L (3.5–5.0 g/dL). Hypocalcemia was defined as a serum calcium level <2.14 mmol/L regardless of the appearance or absence of symptoms of hypocalcemia. In addition to laboratory-verified hypocalcemia, symptoms and signs of hypocalcemia in patients were recorded daily. Recovery of parathyroid function was defined as the return of PTH and serum calcium levels within the reference range without additional calcium or vitamin D replacement. Therapeutic calcium replacement was not prescribed if the patient had no laboratory or clinical signs of hypocalcemia. It was administered to patients with laboratory-confirmed hypocalcemia (serum calcium values <2.00 mmol/L) and patients with symptoms of hypocalcemia regardless of serum calcium laboratory values. Supplementation therapy consisted of orally administered elemental calcium (calcium carbonate, 1-g unit) and/or calcitriol (0.5 microgram unit), with dose adjustment depending on serum calcium values. Calcium gluconate was administered intravenously only if severe symptoms of hypocalcemia were present.

Corrected serum calcium was calculated using Payne's formula [“corrected Ca” (mmol/L) = measured Ca (mmol/L) + 0.020 or 0.025 (40-albumin (g/L))], Clase's formula [“corrected Ca” (mmol/L) = measured Ca (mmol/L) + 0.018 × (35-albumin (g/L))], Jain's formula [“corrected Ca” (mmol/L) = measured Ca (mmol/L) + 0.01 × (30-albumin (g/L))] and Ridefelte's formula [“corrected Ca” (mmol/L) = measured Ca (mmol/L) - 0.0135 × albumin (g/L) + 0.4525]. Patients were discharged on the first or second postoperative day if postoperative calcium and PTH were within the reference range and if the patient's condition was stable and further monitoring of PTH and calcium took place on an outpatient basis. If the patient did not receive replacement therapy during hospitalization, no prophylactic calcium replacement was prescribed after discharge from the hospital.

Statistical data processing uses standard descriptors (arithmetic mean and standard deviations or median). The relationships between the variables were tested using a binary logistic regression model to demonstrate a statistically significant correlation between symptom onset and postoperative hypocalcemia on the fifth day. All tests were performed using a 5% two-way type I error. Each variable that was statistically significantly related to the primary outcome was further analyzed using the ROC (Receiver Operating Characteristic) curve. A cut-off value was determined using the Youden J index (a measure of the sensitivity and specificity of the dichotomous test variable). Area Under the Curve (AUC) >0.6 was considered statistically significant. An additive risk algorithm was then constructed that included variables identified as risk factors for hypocalcemia present on the fifth postoperative day. After calculating the result for all patients, ROC analysis determined its limit value, sensitivity, and specificity. P values ≤0.05 were considered statistically significant. Statistical analysis was performed using MedCalc (Version 11.2.1 © 1993–2010. MedCalc Software bvba Software, Broekstraat 52, 9030 Mariakerke, Belgium) and SPSS (Version 22.0., 2013. IBM SPSS Statistics for Windows, Armonk, NY: IBM Corp.) as well as standard descriptive statistics and frequency tables.

## Results

**Fig. 1:**
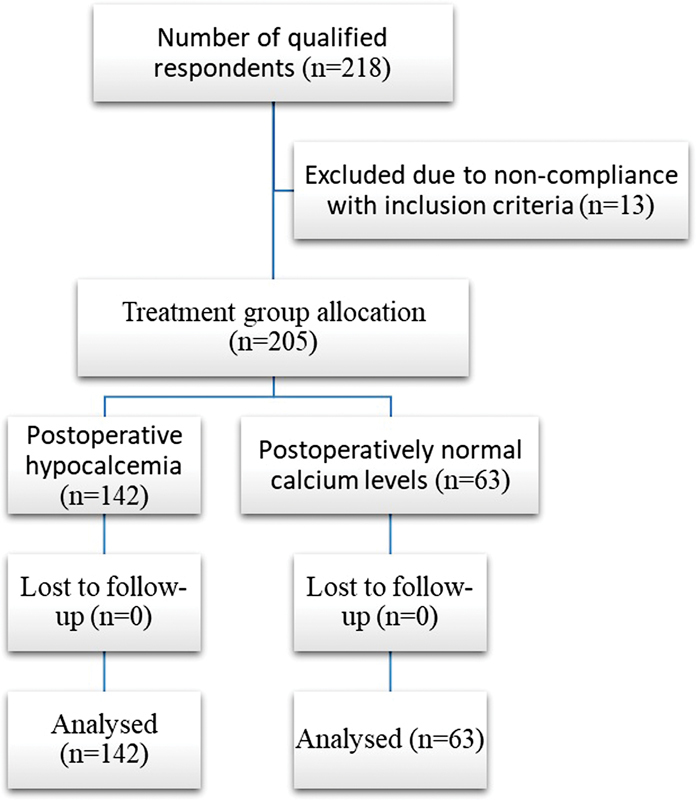
CONSORT flow diagram.

A total of 205 subjects were included in the study, of which 182 were women (88.78%). The mean age was 47.98 years with a standard deviation of ± 15.29 years. The proportion of patients requiring calcium supplementation was 46.34% ( *n* = 95). ( Fig. 1 and Table 1 )

**Table 1:** Clinical and surgical characteristics of 205 patients who underwent total thyroidectomy (Data are expressed as N (number), mean ± SD, or frequency (%))

Patient age (years)	47.98 ± 15.29
Women (N)	182 (88.78%)
Neck dissection (N)	27 (13.17%)
Number of patients needing calcium replacement therapy (N)	95 (46.34%)
PTH on the day of surgery (pmol / L)	3.07 ± 3.03
PTH first postoperative day (pmol / L)	2.65 ± 1.84
Serum calcium on the day of surgery (mmol / L)	2.07 ± 0.28
Serum calcium first postoperative day (mmol / L)	2.00 ± 0.20
Serum calcium on the fifth postoperative day (mmol / L)	2.10 ± 0.10
Albumin (g / L)	45.87 ± 2.57
Ionized calcium on the day of surgery (mmol / L)	1.15 ± 0.10
Ionized calcium on the first postoperative day (mmol / L)	1.2 ± 0.20
Ionized calcium on the fifth postoperative day (mmol / L)	1.2 ± 0.10

The binary logistic regression model showed that performing total thyroidectomy and neck dissection did not impact the occurrence of symptomatic hypocalcemia on the fifth postoperative day concerning patients who did not undergo neck dissection ( *p* = 0.137 and *p* = 0.614). In contrast, the patient's age was strongly associated with the occurrence of symptomatic hypocalcemia, with an odds ratio (OR) of 11.030 ( *p* = 0.001). ROC analysis confirmed that younger age, at a cut-off value of ≤50 years, was associated with a more frequent occurrence of symptomatic hypocalcemia with a sensitivity of 64.21% and a specificity of 51.82%. The majority of patients underwent TT because of confirmed thyroid malignancies or thyroid goiter. In our study, a statistically significant association between hyperthyroidism and the occurrence of symptomatic hypocalcemia on the fifth day after surgery was confirmed by binary logistic regression ( *p* = 0.041), while other thyroid disorders did not affect its occurrence.

While 142 patients (69.3%) were hypocalcemic one hour after surgery with an average serum calcium value of 2.07 ± 0.28 mmol/L, 114 patients (36.4%) were hypocalcaemic with a mean serum calcium level of 2.10 ± 0.10 mmol/L on the fifth postoperative day ( Table 1 ). Only 11 patients had lowered ionized calcium values on the fifth postoperative day with an average value of 1.2 ± 0.10 mmol/L. Mean serum PTH values on the day of surgery were 3.07 ± 3.03 pmol/L, with 49 patients (23.9%) having hypoparathyroidism. Laboratory hypoparathyroidism and hypocalcemia on the day of surgery were recorded in 41 patients, whereas on the first postoperative day, it was present in 38 patients. Clinical hypoparathyroidism, a combination of lower PTH values and symptoms and/or signs of hypocalcemia, was recorded in 31 patients, both on the day of the surgery and the first postoperative day, respectively.

The binary logistic regression model identified age, PTH, calcium, and ionized calcium on the day of surgery, and on the first postoperative day showing statistical significance in predicting symptomatic hypocalcemia on the fifth postoperative day ( Table 2 ).

**Table 2:** Risk analysis of symptomatic hypocalcemia by binary logistic regression

BINARY LOGISTIC REGRESSION	ODDS RATIO (OR)	P VALUE
Age	11,030	0,001
PTH*	11,035	0,001
Serum Ca *	11,609	0,001
Ionised Ca *	5,257	0,022
PTH **	15,058	0,000
Serum Ca **	20,000	0,000
Ionized Ca **	6,147	0,013
Serum Ca ***	17,585	0,000
Payne	11,571	0,001
Clase	11,905	0,001
Jain	11,995	0,001
Ridefelt	12,007	0,001

* concentrations one hour after surgery; ** concentrations on the 1st postoperative day; *** concentrations on the 5th postoperative day.

When analyzing the presence of laboratory-verified hypocalcemia on the fifth postoperative day, hyperthyroidism, PTH, serum calcium one hour after surgery, and PTH alongside serum calcium on the first postoperative day showed statistical significance in predicting biochemically identified hypocalcemia on the fifth postoperative day ( Table 3 ).

**Table 3:** Risk analysis of hypocalcemia on the fifth postoperative day by binary logistic regression

BINARY LOGISTICAL REGRESSION	ODDS RATIO (OR)	P VALUE
Hyperthyroidism	4,162	0,041
PTH *	12,115	0,001
Serum Ca *	6,396	0,011
PTH **	13,717	0,000
Serum Ca **	19,185	0,000
Clase	4,304	0,038
Jain	5,331	0,021
Ridefelt	4,916	0,027

* concentrations one hour after surgery; ** concentrations on the 1st postoperative day.

**Fig. 2:**
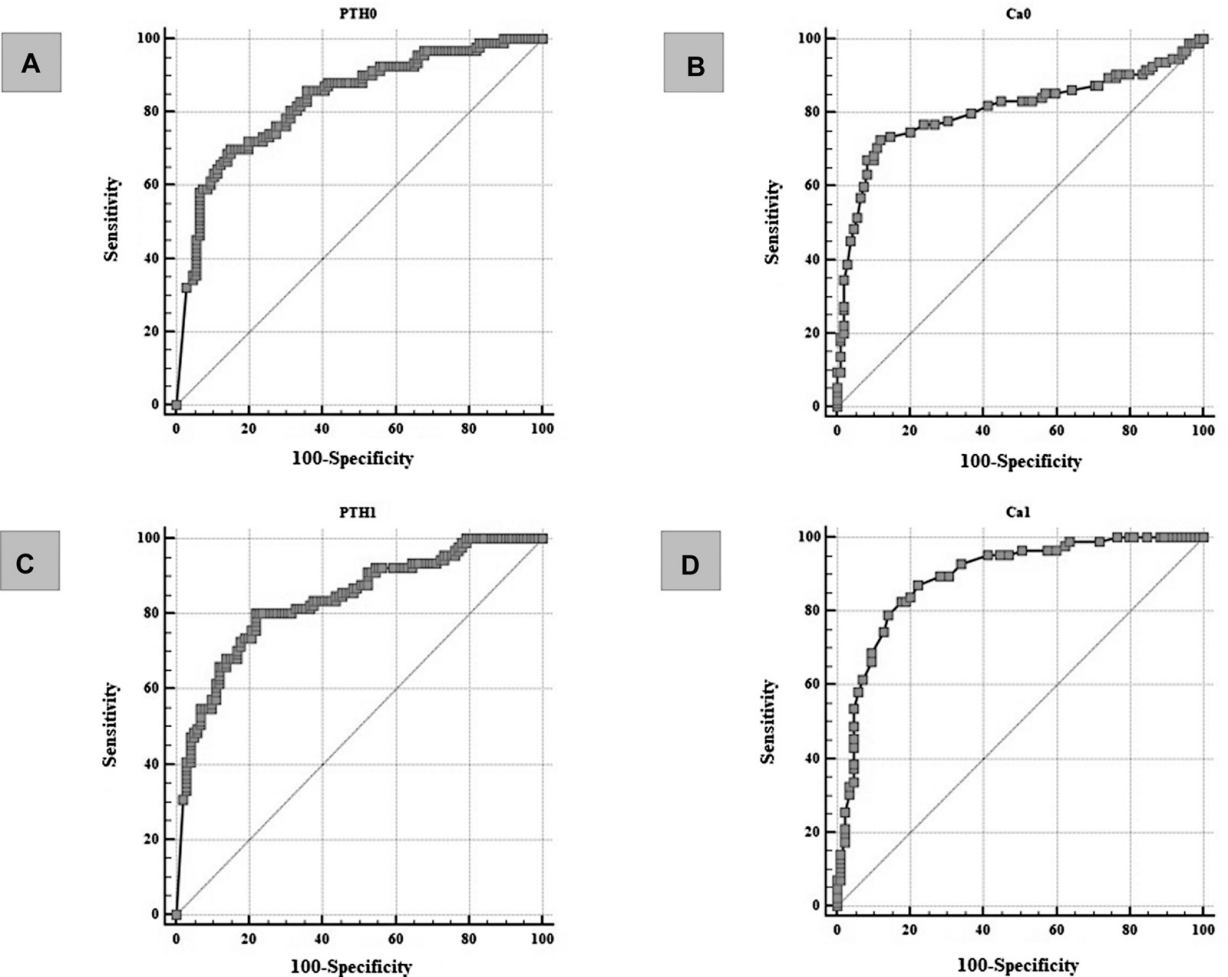
ROC curves of correlations between laboratory parameters and occurrence of symptomatic hypocalcemia. ( **A** ) PTH concentration determined one hour after surgery predicts the onset of hypocalcemia symptoms with a sensitivity of 69.89% and a specificity of 84.91% at a cut-off value of ≤1.51 pmol/L (ROC analysis; AUC 0.832; 95% CI 0.773–0.881; P value <0.0001). ( **B** ) Serum calcium concentration determined one hour after surgery predicts the occurrence of symptomatic hypocalcemia with a sensitivity of 72.63% and a specificity of 88.07% at a cut-off value of ≤2.01 mmol/L (ROC analysis; AUC 0.807; 95% CI 0.746- 0.859; P value <0.0001). ( **C** ) PTH concentration determined on the first postoperative day predicts the occurrence of symptomatic hypocalcemia with a sensitivity of 80.22% and a specificity of 78.22% at a cut-off value of ≤2.03 pmol/L (ROC analysis; AUC 0.835; 95% CI 0.775- 0.884; P value <0.0001). ( **D** ) Serum calcium concentration determined on the first postoperative day predicts the occurrence of symptomatic hypocalcemia with a sensitivity of 79.07% and a specificity of 85.88% at a cut-off value of ≤2.02 mmol/L (ROC analysis; AUC 0.891; 95% CI 0.835–0.934; P value <0.0001).

**Fig. 3:**
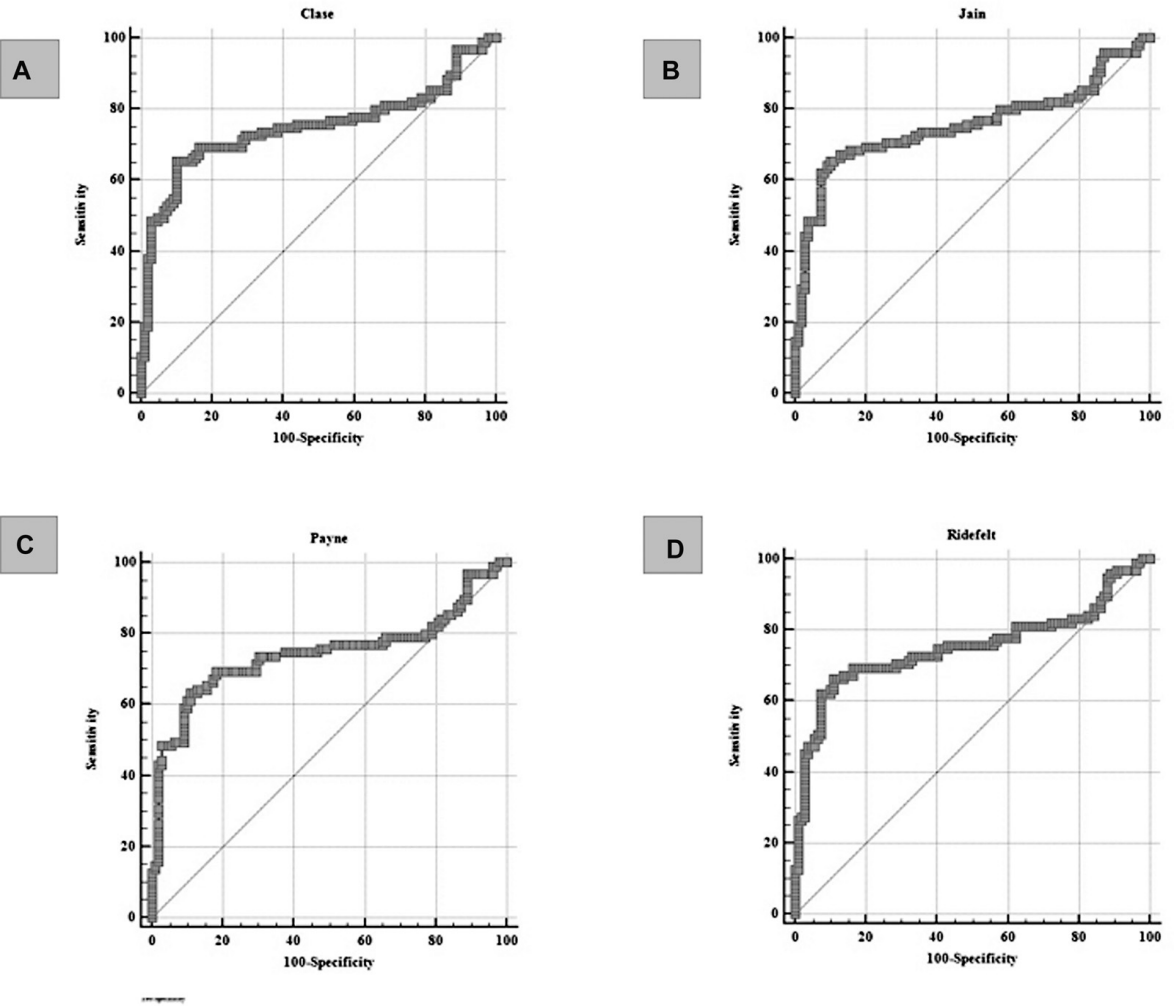
ROC curves of correlations between calcium correction formulas and symptomatic hypocalcemia. ( **A** ) The Clase correction formula predicts the development of symptomatic hypocalcemia with a sensitivity of 65.26% and specificity of 89.91% at the cut-off value of ≤1,801 mmol/L (ROC analysis; AUC 0.751; 95% CI 0.686–0.809; P value <0.0001). ( **B** ) The Jain correction formula predicts the development of symptomatic hypocalcemia with a sensitivity of 65.26% and a specificity of 89.91% at a cut-off value of ≤1.847 mmol/L (ROC analysis; AUC 0.756; 95% CI 0.691–0.814; P value <0.0001). ( **C** ) The Payne correction formula predicts the development of symptomatic hypocalcemia with a sensitivity of 63.16% and specificity of 88.99% at a cut-off value of ≤1.8525 mmol/L (ROC analysis; AUC 0.746; 95% CI 0.680–0.804; P value <0.0001). ( **D** ) The Ridefelt correction formula predicts the development of symptomatic hypocalcemia with a sensitivity of 66.32% and a specificity of 88.99% at a cut-off value of ≤1.8426 mmol/L (ROC analysis; AUC 0.753; 95% CI 0.688–0.811; P value <0.0001).

Statistically significant findings were further evaluated using ROC curve analysis. PTH measured on the first postoperative day had the highest sensitivity (80.2%), whereas calcium sampled one hour after surgery had the highest specificity (88.1%) in predicting symptomatic hypocalcemia ( Fig. 2 ). Clase, Jain, Payne, and Ridefelt's correction formulas were calculated using preoperative albumin and serum calcium values sampled one hour after surgery. When analyzing serum calcium as a predictor of symptomatic hypocalcemia, Ridefelt's correction formula had the highest sensitivity (66%), and the Clase and Jain correction formulas showed the highest specificity (90%) ( Fig. 3 ).

**Fig. 4:**
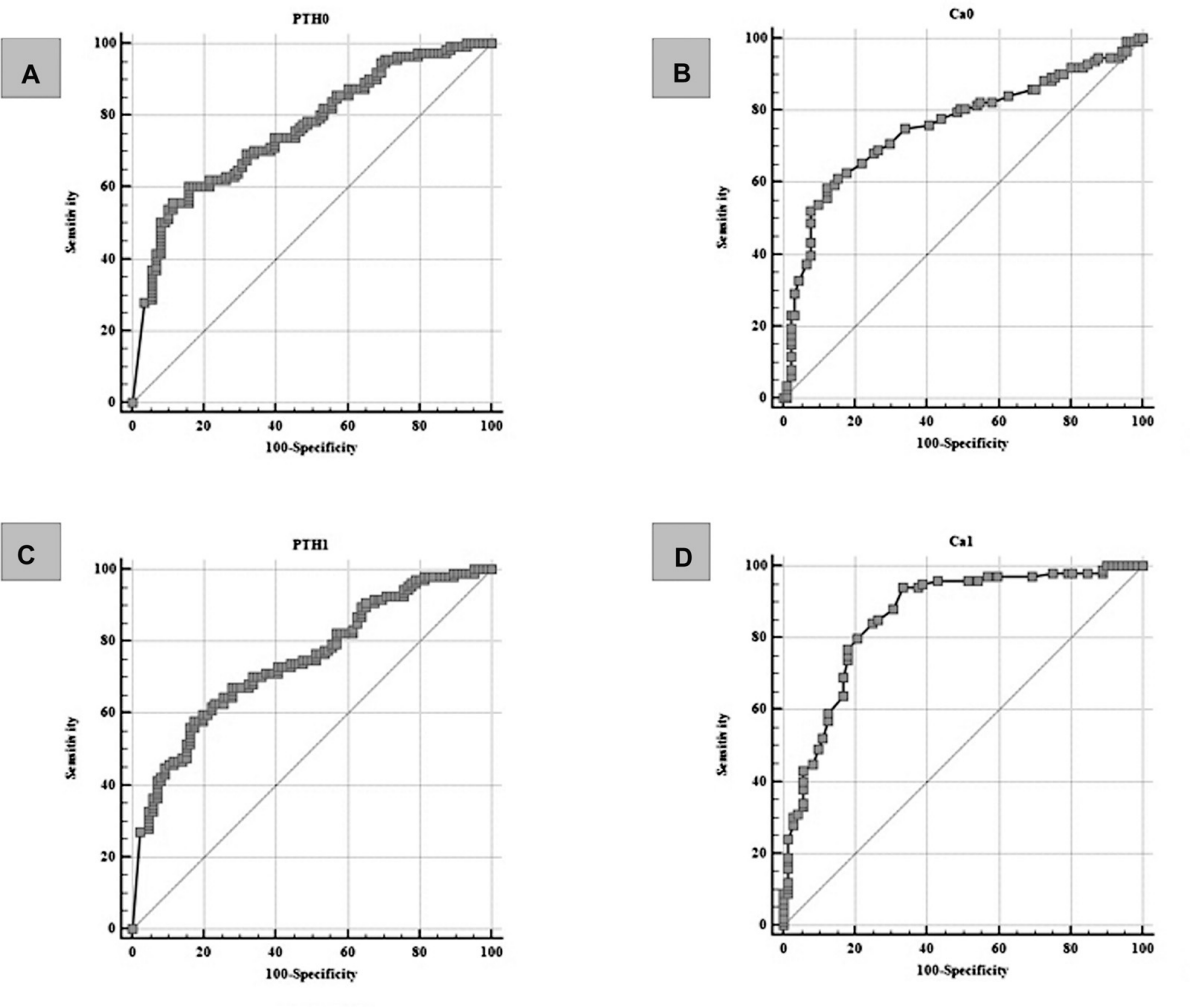
ROC curves of the correlation of laboratory parameters and the occurrence of laboratory verified hypocalcemia on the fifth postoperative day. ( **A** ) PTH concentration determined one hour after surgery predicts the occurrence of hypocalcemia on the fifth postoperative day with a sensitivity of 55.86% and a specificity of 88.64% at a cut-off value of ≤ 1.27 pmol/L (ROC analysis; AUC 0.760; 95% CI 0.695–0.818; *p* < 0.0001). ( **B** ) Serum calcium concentration determined one hour after surgery predicts the occurrence of hypocalcemia on the fifth postoperative day with a sensitivity of 58.41% and a specificity of 87.91% at the cut-off value of ≤ 1.99 mmol/L (ROC analysis; AUC 0.757; 95% CI 0.692- 0.814; *p* < 0.0001). ( **C** ) PTH concentration determined on the first postoperative day predicts the occurrence of hypocalcemia on the fifth postoperative day with a sensitivity of 57.94% and a specificity of 82.56% at a cut-off value of ≤ 1.55 pmol/L (ROC analysis; AUC 0.747; 95% CI 0.679–0.806; *p* < 0.0001). ( **D** ) Serum calcium concentration determined on the first postoperative day predicts the occurrence of hypocalcemia on the fifth postoperative day with a sensitivity of 94% and a specificity of 66.67% at a cut-off value of ≤ 2.1 mmol / L (ROC analysis; AUC 0.860; 95% CI 0.799–0.908; P-value <0.0001).

**Fig. 5:**
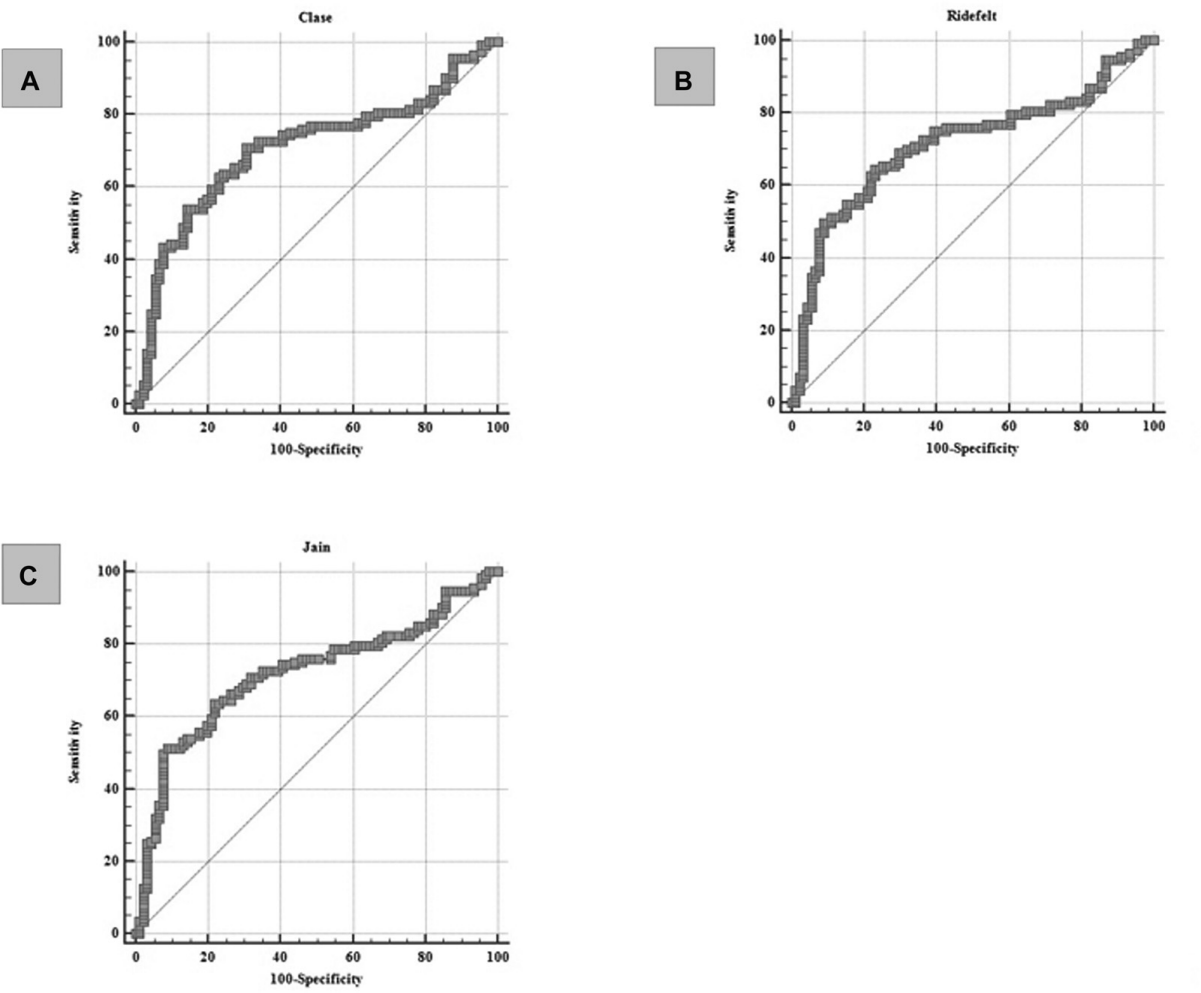
ROC curves of calcium correction formulas predicting the occurrence of laboratory verified hypocalcemia on the fifth postoperative day. ( **A** ) The Clase correction formula predicts the development of hypocalcemia on the fifth postoperative day with a sensitivity of 70.80% and a specificity of 69.23% at the cut-off value of ≤1.8791 mmol/L (ROC analysis; AUC 0.707; 95% CI 0.639–0.768; P value <0.0001). ( **B** ) The Ridefelt correction formula predicts the development of hypocalcemia on the fifth postoperative day with a sensitivity of 64.60% and a specificity of 76.92% at the cut-off value of ≤1.8867 mmol/L (ROC analysis; AUC 0.715; 95% CI 0.647–0.776; *p* < 0.0001). ( **C** ) The Jain correction formula predicts the development of hypocalcemia on the fifth postoperative day with a sensitivity of 51.33% and a specificity of 91.21% at the cut-off value of ≤1,803 mmol/L (ROC analysis; AUC 0.719; 95% CI 0.652–0.780; P value <0.0001).

ROC curve analysis also showed statistically significant associations between the occurrence of laboratory-verified hypocalcemia on the fifth postoperative day and the values of PTH and serum calcium sampled one hour after surgery and on the first postoperative day ( Fig. 4 ). Serum calcium measured on the first postoperative day had the highest sensitivity (94%) in predicting the occurrence of hypocalcemia on the fifth postoperative day, while PTH measured one hour after surgery had the highest specificity (89%). Serum calcium corrected with the Clase formula had the highest sensitivity (71%) in the prediction of hypocalcemia on the fifth day after surgery, and the Jain correction Eq. (91%) showed the highest specificity ( Fig. 5 ).

**Fig. 6:**
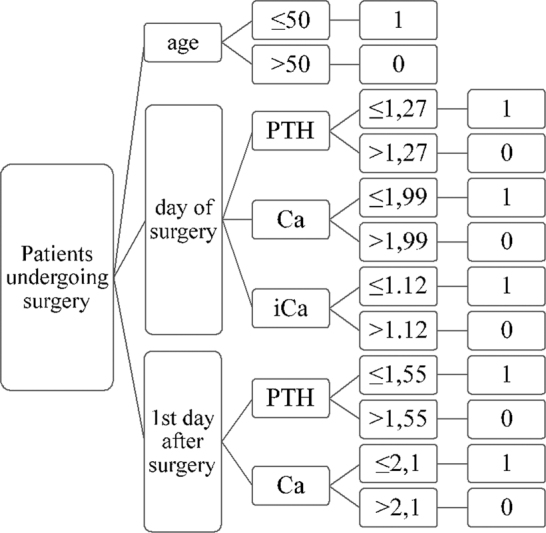
Algorithm for assessing the risk of hypocalcemia on the fifth postoperative day using variables identified in the logistic regression model (age in years, PTH in pmol/L, Ca in mmol/L, iCa in mmol/L).

**Fig. 7:**
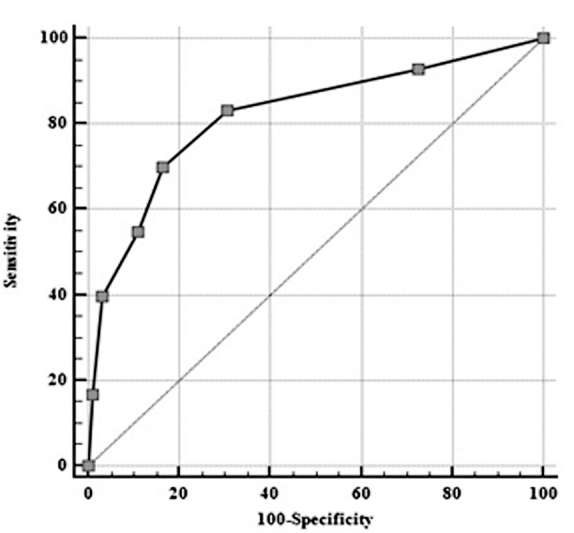
ROC curve of the algorithm assessment of hypocalcemia risk on the fifth postoperative day (ROC analysis; AUC 0.820; cut-off value ≥2; sensitivity 69.91%; specificity 83.52%; 95% CI 0.760–0.870; P-value <.0001).

An algorithm for assessing the risk of hypocalcemia on the fifth postoperative day was constructed by assigning 0 to each variable if its value was under the cut-off value and 1 if its value was over the cut-off value. By summing up the values, a score from 0 to 6 was constructed ( Fig. 6 ). The predictive value of the score was then analyzed about the occurrence of hypocalcemia on the fifth postoperative day. The ROC curve of this derived risk assessment result showed that a value of ≥2 on the first day has a sensitivity of 70% and 83% specificity in identifying patients developing laboratory-verified hypocalcemia on the fifth day ( Fig. 7 ).

## Discussion

The main goal of this study was to determine whether, through the analysis of reproducible, operator-independent data, an accurate assessment of post-thyroidectomy hypocalcemia risk can be made. The secondary goal was to construct and test an algorithm used in predicting postoperative hypocalcemia and compare its accuracy with individual laboratory variables in symptomatic hypocalcemia assessment, thus reducing the likelihood of developing uncomfortable and potentially life-threatening symptoms. Although most cases of hypocalcemia are transient, adequate early risk assessment would have a positive impact on timely treatment with the shortening of hospital stays and cost optimization. 6 The incidence of transient and persistent hypocalcemia is difficult to determine due to the heterogeneity of data in different studies (age, sex, primary diagnosis, location of parathyroid glands). 2 Although some medical institutions tackle postoperative hypocalcemia by administering prophylactic calcium supplements, this practice is still controversial as it can lead to unnecessary over-medication and possible masking of potential complications prior to hospital discharge. 7 12 13

Traditional methods of detecting hypocalcemia include sampling serum and ionized calcium and monitoring for symptoms. A systematic review and meta-analysis study concluded that serum calcium <2.10 mmol/L, sampled 24 hours after surgery, predicted persistent hypocalcemia with a sensitivity of 99%. 9 In addition, one study observed a correlation between predicting future hypocalcemia and the relative serum calcium drop rate postoperatively. 14 Postoperative ionized calcium values of 0.95 mmol/L or lower have also been associated with the occurrence of symptomatic hypocalcemia. 9 15 Although it may seem contradictory to use calcium in determining the prediction of hypocalcemia, its most significant advantage is the ability to predict persistent hypocalcemia, but with low specificity. 9 Despite a positive correlation with symptomatic hypocalcemia, daily sampling of serum calcium values requires prolonged hospitalization. An alternative marker is PTH due to its short half-life (t1/2) of only 3 minutes and significant correlation with the function of the parathyroid glands. 11 One study showed that a drop in PTH >30% 10 minutes after the surgery was linked with an increased risk of postoperative hypocalcemia with sensitivity and specificity over 90%. In the same study, with the help of total serum calcium values with equally high sensitivity and specificity, the development of symptomatic hypocalcemia could be predicted only 12 hours after surgery. 8 Another study found that PTH measured four hours postoperatively was as effective in assessing postoperative hypocalcemia as PTH measured on the first postoperative day in the morning if different laboratory cut-off values for hypoparathyroidism were applied. 16 A systematic review and meta-analysis study found that low PTH concentrations (lower than 6–35 pg / mL), measured one hour to one day after surgery, predicted transient hypocalcemia with a sensitivity ranging from 69 to 100%. 9 A previous study shows that PTH levels <2.9 pmol/L postoperatively were associated with a high risk of hypocalcemia. 17 The heterogeneity of previously listed results is the product of intricate calcium homeostasis with multiple dependent and independent variables affecting postoperative hypocalcemia. 18 In the current study, PTH, serum, and ionized calcium measured one hour after surgery, and PTH and serum calcium measured on the first postoperative day proved to be the most accurate parameters for predicting the development of hypocalcemia on the fifth day after surgery. It is important to apply different cut-off values for the same variables depending on the day to increase the sensitivity and specificity of the test. Lower PTH concentrations successfully identify patients at risk of developing transient hypocalcemia, whereas normal PTH finding excludes the possibility of developing persistent hypoparathyroidism. 19

Due to hypocalcemia being a multifactorial phenomenon, different combinations of variables were used, with the common goal of finding an algorithm that can predict the occurrence of hypocalcemia as early as possible with as much sensitivity and specificity as possible. One study developed an algorithm consisting of preoperative and intraoperative variables (age, sex, thyroid pathology, serum calcium, albumin, alkaline phosphatase blood levels, medications used, the presence of comorbidities, duration of surgery and anesthesia, the volume of intravascularly replenished fluid, the number of parathyroid glands removed, and the type of neck dissection) to determine the length of hospitalization within 24 hours after surgery. 20 Other authors used preoperative calcium concentration <9mg / dL, ultrasound of retrosternal goiter or enlarged thyroid, proven hyper-/hypothyroidism, PTH six hours after surgery <1.58 pmol / L, and Bethesda score ≥4 for the development of the algorithm which can predict the occurrence of hypocalcemia with 100% sensitivity at the expense of lower specificity. (79%) The same study emphasized the need for further evaluation in a more significant number of patients. 21 The third study highlighted preoperative and postoperative serum calcium levels 12 hours after surgery, the number of preserved parathyroid glands, blood vitamin D levels, and nodule size measured during surgery as the most significant variables in predicting postoperative symptomatic hypocalcemia. This algorithm had a sensitivity of 91% and a specificity of 84% but required several tests not routinely performed in patients undergoing total thyroidectomy with the consequent increase in treatment costs and time. 22 The three previous examples show that an algorithm can be constructed using different variables, but the main problem of such algorithms is their complexity and need for additional diagnostic/laboratory tests, which in turn prolong hospitalization stay or increase postoperative cost. In addition, some of the variables used were operator-dependent and represented a significant bias. As seen in Fig. 6 , our algorithm uses objective, user-independent data, which are cost-effective and simple to measure during postoperative care. However, as shown by our data, even though a usable algorithm for predicting hypocalcemia on the fifth postoperative day can be constructed, such a tool is at a disadvantage compared with the combination of PTH and serum calcium values on the first postoperative day considering their specificity and sensitivity. Furthermore, measuring two values instead of five is a simpler and more cost-effective method with a lower possibility of bias. Therefore, using complex algorithms seems impractical in a daily clinical setting.

An additional question concerns using correction formulas in patients with normal serum albumin values. Approximately 50% of calcium is ionized (in its biologically active form), whereas ∼40% is bound to plasma proteins (predominantly albumin), and only ∼10% is in conjunction with anions. The problem with determining the actual calcium concentration occurs in cases of hypo- or hyperalbuminemia. A significant increase or decrease in total serum calcium concentration may occur, while the concentration of ionized calcium remains the same. On the other hand, if the pH of the medium changes, the concentration of ionized calcium changes accordingly. 23 One study found that changing one pH unit changes ionized calcium concentration by as much as 0.36 mmol/L. 24 Since calcium concentrations are subject to significant change, the use of correction formulas may be necessary. 25 Payne's recommendation is to use the formula in case of coexistence of hypocalcemia and hypoalbuminemia, tested by a study in which the use of Payne's formula at albumin values higher than 40 g/L resulted in false low calcium results, i.e., the use of this formula at albumin values higher than 44 g/L decreased the actual calcium value by 0.2 mmol/L. 26 Despite Payne's formula being used the longest, several other correction formulas have been developed more recently: Clase, Jain, and Ridefelt. 27 28 29 All correction formulas were adversely affected by abnormal albumin values, changing pH, and impaired glomerular filtration. In addition, a lower correlation between total serum and corrected calcium was observed. Several studies conclude that the predictive value of ionized rather than albumin-corrected calcium is better in the assessment of patient outcomes. 30 31 32 Another study also confirmed the ineffectiveness of the widespread use of correction formulas by observing a negligible correlation between corrected calcium calculated using the Payne formula and ionized calcium compared with total serum and ionized calcium. 27 30 Our study also showed a better correlation between ionized and serum calcium rather than between corrected calcium and ionized calcium. The explanation for such a phenomenon lies in the complexity of the dynamic equilibrium of ionized calcium, which cannot be correctly predicted using present correction formulas in patients with normal albumin levels.

One of the main limitations of this study is that sampling was done once a day, but more frequent sampling would be uncomfortable for patients. Although our sample size was relatively large, our follow-up was short, impacting the long-term implications of our results. The advantage of this study is the comprehensiveness of the factors predicting the occurrence of symptomatic hypocalcemia while accounting for results analyzed with regard to both symptomatic and laboratory-verified hypocalcemia, alongside verifying the need for calcium correction formulas and reliability of derived risk scoring on a single cohort of patients.

## Conclusions

This prospective study confirmed the possibility of early prediction of symptomatic and laboratory-verified hypocalcemia occurrence after total thyroidectomy and the identification of low-risk patients who are candidates for safe hospital discharge. The most sensitive predictor of symptomatic hypocalcemia present on the fifth postoperative day was PTH sampled on the first postoperative day. The combination of PTH and total serum calcium sampled on the first postoperative day had the highest sensitivity and specificity, with cut-off values of ≤1.55 pmol/L and ≤2.1 mmol/L, respectively. The need for algorithms and correction formulas is limited.
